# Epidemiological characteristics of pesticide poisoning in Jiangsu Province, China, from 2007 to 2016

**DOI:** 10.1038/s41598-019-44986-7

**Published:** 2019-06-13

**Authors:** Ning Wang, Qingtao Jiang, Lei Han, Hengdong Zhang, Baoli Zhu, Xin Liu

**Affiliations:** 1 School of Public Health, Southeast University, Nanjing, 210009 Jiangsu China; 2 Department of Prevention and Control for Occupational Disease, Jiangsu Provincial Center for Disease Prevention and Control, Nanjing, 210009 Jiangsu China; 3 Department of Medicine, Jiangsu Health Vocational College, Nanjing, 210029 Jiangsu China

**Keywords:** Health occupations, Health occupations, Health occupations, Risk factors, Risk factors

## Abstract

To investigate the characteristics of pesticide poisoning in Jiangsu province and provide a scientific basis for measures for the prevention of pesticide poisoning. Information from pesticide poisoning report cards from 2007 to 2016 was derived from the Occupational Disease and Occupational Health Information Monitoring System, and the characteristics of pesticide poisoning patients were assessed via descriptive analysis. A total of 30,789 pesticide poisoning cases were reported in Jiangsu Province. Of these cases, 23,557 were non-occupational poisonings, accounting for 76.5% of the reported cases. There were 1705 deaths from pesticide poisoning, and the fatality rate was 5.5%. Numerous cases occurred in northeast Jiangsu. Pesticide poisoning cases were primarily concentrated in individuals 36–60 years of age. Pesticide poisoning primarily occurred in the summer. The top types of pesticides causing poisoning were organophosphates. The fatality rate of intentional pesticide poisoning was the lowest among the age group of 0 to 14 years, while the highest was noted among people over 61 years. Therefore, pesticide poisoning is a major problem in Jiangsu Province. We need to strengthen the management of highly toxic pesticides and implement effective health education on the use of pesticides.

## Introduction

Pesticides are a large group of heterogeneous chemicals that include a wide range of products, such as insecticides, herbicides, fungicides, and rodenticides^1^. Different types of pesticides are used to control and repel pests in different types of fields^2^. The benefits of pesticides have come at a cost, and their continued use is the frequent subject of debate^3^. In developing countries, where there are incomplete regulations, a lack of monitoring systems, low enforcement, lack of training, inadequate access to information systems, poorly managed or non-existent personal protective equipment, and large agricultural-based populations, the incidence of pesticide poisoning is expected to be higher than in developed countries^4,5^. Furthermore, more than 168,000 people die from pesticide suicide every year, the majority of whom are from developing countries^6^. Pesticide poisoning could be divided into occupational and non-occupational pesticide poisoning^7,8^. Pesticides are dangerous to use and poorly managed. Due to their easy access and widespread application in agriculture, death by pesticide consumption is an extremely universal approach to suicide^9^. Pesticide self-poisoning accounts for one-third of the total number of suicides worldwide. It is estimated that there are 258,000 suicides per year^10^.

China has a large amount of pesticides, consuming approximately 1.4 million tons per year. With the development of agriculture, the production and varieties of pesticides are increasing^11^. However, when pesticides are applied to increase and protect production, improper use or misuse can induce severe consequences. Previous studies have shown that taking high levels of pesticides is a common method of suicide in rural China. Studies estimate that over 160,000 people commit suicide every year in China^7,8^. Special attention has been paid to the fact that two-thirds of hospitalizations and the majority of deaths were due to intentional self-poisoning rather than occupational or accidental poisoning. To reduce intentional and unintentional deaths from pesticide poisoning, the Chinese government has conducted on-going epidemiological surveillance of pesticide use and monitored pesticide poisoning in communities and hospitals. Since 2006, Jiangsu Province has established the Occupational Diseases and Occupational Health Information Monitoring System (ODSRS), an internet-based reporting system that collects information on all types of occupational hazards and occupational poisoning^11^.

To further understand the current situation, pathogenesis, characteristics and distribution of pesticide poisoning in Jiangsu, we obtained detailed data on pesticide poisoning in Jiangsu Province from 2007 to 2016 through the ODSRS for a thorough and descriptive analysis. These findings could be used to encourage the development and implement a strategy for the safe handling, use and disposal of pesticides and reduce the incidence of pesticide poisonings.

## Results

### Basic information on pesticide poisoning

From 2007 to 2016, there were 30,789 cases of pesticide poisoning in Jiangsu Province. Among these cases, 12,867 involved males, and 17,922 involved females. In total, 1705 cases ended in death, yielding a mortality rate of 5.5%, including 765 males and 940 females. The number of deaths attributed to non-occupational pesticide poisoning was 1,666, which led to a fatality rate of 7.1%. The total mortality rate of male pesticide poisoning was significantly higher than that of female pesticide poisoning (χ^2^ = 6.28, *P* < 0.05) (Table 1).

**Table 1:** Annual reported pesticide poisoning cases in Jiangsu Province.

Year	Incident Number			Death Number			Fatality Rate (%)			P*
	Male	Female	Total	Male	Female	Total	Male	Female	Total	
2007	2688	3678	6366	142	180	322	5.3	4.9	5.1	
2008	2022	2985	5007	125	162	287	6.2	5.4	5.7	
2009	1617	2345	3962	102	125	227	6.3	5.3	5.7	
2010	1532	2159	3691	87	115	202	5.7	5.3	5.5	
2011	1252	1930	3182	76	108	184	6.1	5.6	5.8	
2012	1086	1466	2552	58	69	127	5.3	4.7	5.0	
2013	881	1184	2065	50	57	107	5.7	4.8	5.2	
2014	586	770	1356	30	43	73	5.1	5.6	5.4	
2015	614	666	1280	61	44	105	9.9	6.6	8.2	
2016	589	739	1328	34	37	71	5.8	5.0	5.3	
Total	12867	17922	30789	765	940	1705	5.9	5.2	5.5	< 0.05

*Two-sided χ^2^ test.

### Regional distribution of pesticide poisoning

**Figure 1:**
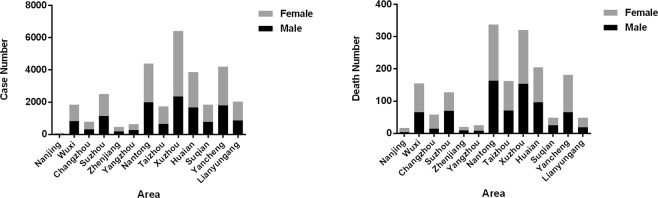
(**A**) and (**B**) are the regional distribution of reported cases and the deaths by pesticide poisoning, respectively, in Jiangsu Province from 2007 to 2016.

Compared with occupational pesticide poisoning, the number of cases, number of deaths and mortality rate of non-occupational pesticide poisoning are relatively high, which indicates that the main problem of pesticide poisoning in Jiangsu Province is non-occupational pesticide poisoning. The most ubiquitous cases of pesticide poisoning occurred in Xuzhou, Nantong, and Yancheng. The number of cases of pesticide poisoning in Nanjing was the lowest (73 cases), comprising 0.2% of all cases in Jiangsu Province. In addition, 8 cases of pesticide poisoning were unknown. The top three pesticide poisoning deaths occurred in Nantong, Xuzhou and Huai’an (Table 2 and Fig. 1).

**Table 2:** Regional distribution of pesticide poisoning cases in Jiangsu Province.

Variable		Incident Number			Death Number			Fatality Rate (%)			P*
		Male	Female	Total	Male	Female	Total	Male	Female	Total	
Area											< 0.001
Southern	Nanjing	28	45	73	4	13	17	14.3	28.9	23.3	
Jiangsu	Wuxi	819	1023	1842	65	90	155	7.9	8.8	8.4	
	Changzhou	314	467	781	15	43	58	4.8	9.2	7.4	
	Suzhou	1140	1365	2505	70	57	127	6.1	4.2	5.1	
	Zhenjiang	183	272	455	9	11	20	4.9	4	4.4	
Northern	Yangzhou	270	360	630	8	17	25	3	4.7	4	
Jiangsu	Nantong	1981	2420	4401	163	175	338	8.2	7.2	7.7	
	Taizhou	657	1077	1734	71	91	162	10.8	8.4	9.3	
	Xuzhou	2357	4063	6420	154	167	321	6.5	4.1	5	
	Huai’an	1667	2193	3860	96	109	205	5.8	5	5.3	
	Suqian	777	1064	1841	25	23	48	3.2	2.2	2.6	
	Yancheng	1812	2382	4194	66	115	181	3.6	4.8	4.3	
	Lianyungang	859	1186	2045	19	29	48	2.2	2.4	2.3	
Unknown		3	5	8	0	0	0	0	0	0	
Total		12867	17922	30789	765	940	1705	5.9	5.2	5.5	

*Two-sided χ^2^ test.

### Distribution of the types of pesticides causing poisoning

The pesticides causing poisoning can be roughly classified into seven categories: organic phosphates, carbamates, halogenated insecticides, other insecticides, herbicides and fungicides, rodenticides and other pesticides. The top three pesticides causing poisoning were organophosphates, herbicides and fungicides, and other insecticides. The number of poisonings by organophosphate pesticides was the highest (18,548), accounting for 60.2% of all cases, followed by 3,595 herbicides and fungicides (11.7%) and 2,703 other insecticides (8.8%). Among the organophosphate pesticides, methamidophos and dichlorvos resulted in the most pesticide poisoning cases. Herbicide and fungicide pesticide poisoning was primarily focused on paraquat and other herbicides (Table 3). The pesticide with the highest rate of poisoning was paraquat, which also had the highest fatality rate (11.6%). Among the seven categories, organophosphate pesticides triggered the highest (6.5%) fatality rate (χ^2^ = 942.10, *P* < 0.001).

**Table 3:** Pesticide types involved in pesticide poisoning in Jiangsu Province.

Type	Incident Number	Death Number	Fatality Rate (%)	P*
**All**	30789	1705	5.5	<0.001
**Organophosphates**	18548	1204	6.5	
Dichlorvos	4521	267	5.9	
Methamidophos	6687	560	8.4	
Parathion	1010	39	3.9	
Omethoate	2205	177	8.0	
Trichlorfon	247	6	2.4	
Isocarbophos	75	4	5.3	
Other organophosphates	3803	151	4.0	
**Carbamates**	1936	32	1.7	
Carbofuran	355	16	4.5	
Methomyl	757	2	0.3	
Other carbamates	824	14	1.7	
**Halogenated insecticides**	1333	16	1.2	
Fluoroacetamide	27	0	0.0	
Other halogenated insecticides	1306	16	1.2	
**Other insecticides**	2703	159	5.9	
Organochlorine	219	12	5.5	
Chlordimeform	212	5	2.4	
Dimehypo	478	44	9.2	
Other insecticides	1794	98	5.5	
**Herbicides and fungicides**	3595	158	4.4	
Paraquat	993	115	11.6	
Other herbicides	2203	39	1.8	
Fungicides	399	4	1.0	
**Rodenticides**	791	33	4.2	
Tetramine	155	12	7.7	
Anticoagulant rodenticides	158	2	1.3	
Other rodenticides	478	19	4.0	
**Others**	1883	103	5.5	
Multipurpose formulation	532	14	2.6	
Biochemical pesticide	226	11	4.9	
Others unspecified	1125	78	6.9	

*wo-sided χ^2^ test.

### Seasonal distribution of pesticide poisoning

**Figure 2:**
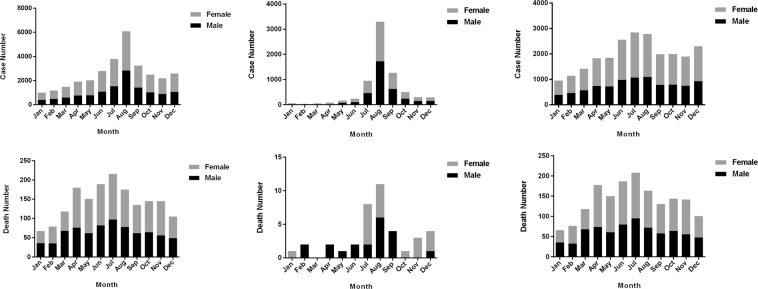
The number of pesticide poisoning cases and deaths in different months from 2007 to 2016 in Jiangsu Province. (**A**,**D**) are the total number of pesticide poisoning cases and deaths in different months; (**B**,**C**) are the cases of occupational and non-occupational pesticide poisoning in different months. (**E**,**F**) are the deaths of occupational and non-occupational pesticide poisoning in different months.

Pesticide poisoning occurred throughout the year during the study period in Jiangsu Province. The highest number of reported cases occurred in summer, with 12.3% and 19.8% of all cases occurring in July and August, respectively. The lowest number of cases occurred in January (3.3%), as shown in Fig. 2A. Occupational pesticide poisoning occurred most often in the summer, with 8190 cases representing 34.8% of the total occupational pesticide poisoning cases (Fig. 2B). Meanwhile, the cases of non-occupational pesticide poisoning were primarily concentrated in summer, with 5505 cases constituting 76.1% of all non-occupational pesticide poisoning cases (Fig. 2C). Total pesticide poisoning cases were highest in July and lowest in January (Fig. 2D). A similar distribution was observed for deaths due to non-occupational pesticide poisoning (Fig. 2E). Deaths induced by occupational pesticide poisoning were highest in August and lowest in March (Fig. 2F).

### Age distribution of pesticide poisoning cases

Pesticide poisoning cases were predominantly concentrated in the age group of 15–60 years. The number of cases in the age group of 36–60 years was the highest, accounting for 47.9% of all cases (Table 4). Occupational pesticide poisoning cases were primarily found in the age group of 36–60 years, and the number of cases in this population accounted for 65.4% of all occupational poisoning cases. Deaths induced by pesticide poisoning were concentrated in people over 36 years old, most of whom were older than 61 years, accounting for 46.9% of all deaths. Fewer deaths caused by pesticide poisoning were noted in the group less than 35 years of age, only representing 14.1% of total deaths. Comparably, a distribution pattern was found in non-occupational pesticide poisoning and reported deaths from occupational pesticide poisoning. Among the number of intentional pesticide poisoning deaths in different age groups, the lowest number occurred in people 0–14 years old, and the highest occurred in people over 61 years old. The number of intentional pesticide poisoning cases increased gradually with age. There were significant differences in the distribution of mortality among different age groups (χ^2^ = 540.84, *P* < 0.001).

**Table 4:** Reported cases and deaths of pesticide poisoning in different age groups.

Variable	Occupational Pesticide Poisoning			Non-occupational Pesticide Poisoning			P*
	Incident number	Death number	Fatality (%)	Incident number	Death number	Fatality (%)	
Age							<0.001
0–14	31	0	0	1107	15	1.36	
15–35	672	9	1.34	7154	217	3.03	
36–60	4730	13	0.27	10013	651	6.50	
61-	1799	17	0.95	5283	783	14.82	
Total	7232	39	0.54	23557	1666	7.07	

*Two-sided χ2 test.

### Stratified analysis of pesticide poisoning in different economic conditions

Based on the above studies, we found significant differences in pesticide poisoning populations based on age (*P* < 0.001) and sex (*P* < 0.05). Age and sex may be mixed factors, affecting the overall effect of the region in the preliminary analysis. Therefore we use age and sex as factors and stratified Chi-square tests to analyse the impact of regional factors on the risk of disease at different ages and genders. We divided the study population in southern and northern Jiangsu into different subgroups according to age and sex (Table 5). For people aged 36–60 and over 61 years, a significant difference in pesticide poisoning was noted between northern and southern Jiangsu (OR = 0.69, 95% CI = 0.57–0.83, *P* < 0.001; OR = 0.78, 95% CI = 0.65–0.92, *P* < 0.001). Similarly, female pesticide poisoning in northern and southern Jiangsu exhibited statistical significance (OR = 0.72, 95% CI = 0.61–0.84, *P* < 0.001).

**Table 5:** Stratified analysis of pesticide poisoning in different economic conditions in Jiangsu Province.

Variables	Northern Jiangsu			Southern Jiangsu			P*	OR (95% CI)
	Incident	Death	Fatality (%)	Incident	Death	Fatality (%)		
**Age**								
0–14	27	0	0	4	0	0	0.22	4.35 (0.57–33.20)
15–35	590	8	1.36	82	1	1.22	0.62	1.09 (0.79–1.53)
36–60	4242	9	0.21	488	4	0.82	<0.001	0.69 (0.57–0.83)
61-	1544	15	0.97	255	2	0.78	<0.001	0.78 (0.65–0.92)
**Sex**								
Male	10384	603	5.81	2483	162	6.56	0.185	0.89 (0.74–1.06)
Female	14751	726	4.92	3171	214	6.75	<0.001	0.72 (0.61–0.84)

*Two-sided χ2 test.

## Discussion

The results of the reported pesticide poisoning cases from 2007 to 2016 showed that the incidence of pesticide poisoning in Jiangsu Province was decreasing. One study found that the health effects of a more comprehensive ban on highly toxic pesticides is needed to create a balance between increased agricultural costs and reduced health care costs and deaths^12^. The number of reported cases and mortality of non-occupational pesticide poisoning are higher than those of occupational pesticide poisoning, which indicates that the primary problem of pesticide poisoning in Jiangsu is non-occupational in origin. This finding is consistent with the conclusions found in similar Zhejiang Province. The two provinces both mainly plant rice and have consistent poisoning from the same pesticides. The rate of consumption of insecticides is the largest, followed by herbicides and rodenticides, while organophosphorus insecticides, pyrethroids and other insecticides are the main pesticides. Non-occupational poisoning is mainly triggered by suicide or accidental ingestion^5,13^. Given that non-occupational pesticide poisoning is easily detected, the case fatality rate is correspondingly high. However, occupational pesticide poisoning mostly occurs at occupational sites and workers are more likely to receive timely treatment after poisoning. Thus, the mortality of occupational pesticide poisoning is accordingly maintained at a low level. There are also reported sex differences in pesticide poisoning^14^. Women’s vulnerability to pesticides may have psychological, behavioural and socio-economic roots. In addition, women have a lower proportion of self-protective behaviours than man when applying pesticide^15^. In the light of the facts outlined above, sex-related working conditions may increase pesticide exposure among females^16^.

This study revealed that the use of pesticides in Jiangsu Province was ubiquitous, and there were reports of pesticide poisoning cases in various cities. Reportedly, the case fatality of pesticide poisoning in the developing world is higher than Western countries, which is attributed to the following reasons: the high toxicity of locally available poisons, the difficulty of transporting patients to the hospital over long distances, the lack of health care workers compared with the large number of patients, and the lack of facilities and antidotes^17^. The occurrence of pesticide poisoning has certain regional differences. The number of pesticide poisoning cases in northern Jiangsu exceeds that in southern Jiangsu, which may be relevant to the economic base and the poor level of industrial and agricultural development in the region^2,11^. Accordingly, it is important to optimize the control of pesticide hazards in northern Jiangsu.

The study found that from 2007 to 2016, the cases of organophosphate pesticide poisoning in Jiangsu were the highest, among which dichlorvos, methamidophos and omethoate were principal culprits. Among the seven classes of pesticides, the class that contributed to the greatest mortality rate was organophosphate pesticide and the specific agent that led to the highest mortality was paraquat, which is consistent with the conclusions of Roberts MD^4^. This finding illustrates that the safe use and management of organophosphate pesticides should be strengthened at the peak of their usage, and the application of highly toxic pesticides, such as paraquat, should be given more attention^4,12^. In terms of the age of those affected by occupational or non-occupational pesticide poisoning, individuals 36–60 years old were primarily affected although the mortality rate was highest in people over 60 years old. Studies have shown that socially disadvantaged groups are at a higher risk of pesticide poisoning^18^. With economic development, increasing number of adolescents are gradually leaving rural areas and entering the city to work^19^. Thus, the aged have consequently become the main farming work force, and there are more opportunities for pesticide contact. Meanwhile, as individuals grow older, their physical function and resistance decrease. Additional factors include depression and other illnesses which can lead to suicide by oral pesticides in the elderly^20^. Hence, to reduce the occurrence of occupational pesticide poisoning, it is critical to conduct health education for middle-aged and elderly people, publicize the correct use of pesticides and spraying methods, and focus on the utilization of personal protective devices when spraying high-toxic pesticides^21^.

The seasonal distribution of pesticide poisoning indicates that there are significant temporal changes in occupational pesticide poisoning in Jiangsu, which mainly occurred from July to September, accounting for 76.1% of all occupational pesticide poisoning cases. This finding is also consistent with the conclusions of Zhejiang^11^. This seasonal increased use of pesticides may be relevant to a higher pest burden during summer and autumn leading to an increased need and subsequent use in the amount pesticide application. The higher temperature in summer and autumn increases the volatilization of pesticides and enhances the chance of pesticide exposure. Meanwhile, farmers lack training in protection knowledge, lack self-protection awareness, and do not wear individual protective equipment as required. Therefore, in summer and autumn, it is imperative to promote the standardization of the production, transportation and use of pesticides and stress the importance to personal protection. There are more female cases of occupational pesticide poisoning than male cases, which is different from previous reports^18,21^. It is possible that more male migrant workers are located in rural areas of Jiangsu.

Studies have presented that the absolute number of suicide deaths of China ranks first in the world, and pesticide poisoning ranks first among methods of suicide^6^. According to the data, the number of cases of intentional pesticide poisoning accounted for 64.8% of the total number of pesticide poisoning cases, and the number of intentional pesticide poisoning deaths constituted 93.1% of all deaths. This study also found that the case fatality rate of intentional pesticide poisoning increased by age, especially in the elderly over 60 years. The mental emptiness and physical diseases caused by the “empty nest” in elderly populations are the main factors of suicide^22,23^. Furthermore, elderly individuals generally exhibit poor health, more comorbidities^21^ and poor prognosis, resulting in higher mortality^24^. We should take comprehensive preventive measures with the participation of the whole society and coordination of various departments to strengthen health education, psychological counselling and scientific propaganda of first aid for poisoning and focus on prevention and education of high-risk groups of suicide.

Most cities in southern Jiangsu are coastal cities, and the transportation industry is more developed compared with that in northern Jiangsu. Agriculture is also an important reason for the economic disparity between northern and southern of Jiangsu Province. Most of the reported cases of pesticide poisoning in Jiangsu Province from 2007 to 2016 occurred in the north and particularly included women and elderly individuals. This information is related to the development of industry and agriculture and the low economic level in northern Jiangsu. The reason may be that in the economically underdeveloped northern Jiangsu, women and the elderly are mostly engaged in agricultural work. Pesticide poisoning not only represents a large economic burden and psychological pressure to farmers but also a waste of medical and health resources and human resources. The government and relevant departments should increase their economic input to publicize measures for the prevention and control of pesticide poisoning.

The advantage of this research is that it includes pesticide poisoning cases from Jiangsu Province regardless of age. Many studies have focused exclusively on middle-aged individuals and paid little attention to pesticide poisoning in children and the elderly. In addition, this study focused on the incidence of pesticide poisoning in Jiangsu Province in the past decade, and the number of cases is relatively large and representative. The limitation of this study is that the number of pesticide poisoning cases is potentially underestimated.

The development of highly effective and low-toxic pesticides should be encouraged. Moreover, the population should be educated on safe production and use of pesticides, detoxification knowledge and personal protection capabilities^9–12,24^. Organophosphorus pesticides are the most commonly used drugs for suicide via oral ingestion and are the focus of suicide prevention. It is necessary to strengthen the knowledge of emergency rescue workers in primary medical institutions on pesticide poisoning. Moreover, government departments should regularly provide mental health education.

In summary, this paper described the situation of pesticide poisoning in Jiangsu Province from 2007 to 2016. The results indicated that the situation of non-occupational pesticide poisoning in Jiangsu Province remains grim, especially for intentional pesticide poisoning, which causes high mortality in elderly individuals. More measures should be taken to protect individuals from pesticide poisoning.

## Methods

### Ethics

All the informational data used in our study were encrypted and obtained from the official pesticide poisoning statistics and the ODSRS by the Jiangsu Provincial Centre for Disease Control and Prevention (JSCDC), China. The private information related to pesticide poisoning was encrypted. Our study complied with the Declaration of Helsinki and was exempted from institutional ethical review by the Research Ethics Board of JSCDC.

### Data source

The pesticide poisoning data were mainly obtained from the ODSRS in Jiangsu Province from January 1, 2007 to December 31, 2016. The ODSRS includes data collected from hospitals, community health service centres, and clinical institutions. The data is entered into an online centralized system. According to the requirements of the system report, after receiving and treating the pesticide poisoning patients at all levels of health institutions, the attending physicians should complete the report card of pesticide poisoning and report the incident through the network within the prescribed time. Per the information included in the pesticide poisoning report card, sex, age, region, poisoned pesticide name, poisoning date, application method, diagnostic institution, poisoning reason, and date of diagnosis of the pesticide poisoning patient should be collected. the collected data is limited and does not include treatment and only reports a qualitative description of recover, improvement or death.

### Statistical analysis

The data of pesticide poisoning in Jiangsu Province from 2007 to 2016 were introduced into EXCEL 2007 for sorting, and GraphPad Prim 5 was used for plotting. Data management and analysis were performed using SPSS 20.0. Qualitative data were described by relative numbers. The mortality of pesticide poisoning population was analysed using the chi-square test to determine significant differences between two or more groups. *P*-values < 0.05 signified statistical significance.

## Data Availability

Data supporting the findings of the current study are available from the corresponding author on reasonable request.
