# Publisher Correction: Epidemiological characteristics of pesticide poisoning in Jiangsu Province, China, from 2007 to 2016

**DOI:** 10.1038/s41598-020-58044-0

**Published:** 2020-01-31

**Authors:** Ning Wang, Qingtao Jiang, Lei Han, Hengdong Zhang, Baoli Zhu, Xin Liu

**Affiliations:** 1 School of Public Health, Southeast University, Nanjing, 210009 Jiangsu China; 2 Department of Prevention and Control for Occupational Disease, Jiangsu Provincial Center for Disease Prevention and Control, Nanjing, 210009 Jiangsu China; 3 Department of Medicine, Jiangsu Health Vocational College, Nanjing, 210029 Jiangsu China

Correction to: *Scientific Reports* 10.1038/s41598-019-44986-7, published online 13 June 2019

The original version of this Article contained a typographical error in the spelling of the author Ning Wang in the equal contribution statement, which was incorrectly given as Ning Wanga. This error has now been corrected in the HTML and PDF versions of this Article.

